# Observations on the Puerperal Fevers Which Occurred at the Maternité of Paris in 1829; on the Various Modes of Treatment That Were Adopted, and Particularly on Mercurials, Emetics, and Bloodletting

**Published:** 1830-11

**Authors:** M. Tonnelle


### PUERPERAL FEVERS.

*Observations on the Puerperal Fevers which occurred at the Maternité of Paris in* 1829*; on the various Modes of Treatment that were adopted, and particularly on Mercurials, Emetics, and Bloodletting.* By M. Tonnelle.*

(Continued from our October Number, p. 314.)

*Treatment of Puerperal Fevers.* It must be evident that no exclusive treatment can be laid down for a disease which assumes so many different forms: and still, if we refer to writers, we find that most of them have a particular mode of practice, which they consider the only one that ought to be employed. Some adopt general, while others trust to local bleeding. By some the employment of mercury is advised, and by others it is entirely proscribed. Emetics and purgatives are also warmly recommended by one party, and as strongly opposed by another. These opposite and exclusive opinions are founded upon some narrow systematic idea, which is maintained in one day, and forgotten the next. An exclusive method of treatment is supported upon a few insulated facts. Certain therapeutic means, the utility of which depended upon a particular form or stage of the disease, or upon certain seasons or conditions of the atmosphere, are thought to be generally applicable. The remedy is highly praised, and put to the test of further trial; but, as the circumstances which originally rendered it successful are no longer present, it of course fails, and it is given up as precipitately as it was at first adopted. Ipecacuanha may be adduced as an example; and many other remedies, after having been in much repute for a certain time, are deprecated, because they have been misapplied, and hence are undeservedly neglected. M. Desormeaux has taken a wider and more philosophic view of the treatment of puerperal fevers. The various modes of practice about to be described were not considered by him to possess a fixed and absolute value. Their utility is relative and subordinate to the different forms of the disease, and to the different conditions of the atmosphere which sensibly influence the malady. All we can do, in the present state of our knowledge, is to record the remedies that have succeeded at one period in certain well-defined cases, and also those which have failed. By numerous cases thus selected, at different times and in various circumstances, we may at length be enabled to establish a well-founded system, which in the present day is impossible.

*Of general Bleeding.* This means may either be eminently useful or prejudicial, according to the period when it is employed, and especially according to the species of the disease. M. Desormeaux only advised general bleeding in those cases of the pure inflammatory form, which have been before described, and in such instances, which were not very common, great advantage was derived from the lancet. When boldly employed from the beginning, and repeated several times in twenty-four hours, general bleeding sometimes cut short the disease, or at least diminished its severity, and hastened its termination: but more commonly the general symptoms were alone relieved, and other means were required to combat the local inflammation. Venesection was rarely beneficial in the second stage of the disease, but still it was sometimes occasionally useful, when, after suppuration and effusion, there was strong and general reaction, with a hard and quick pulse, hot skin, red face, and animated expression of the eyes. Under these circumstances the appearance of some secondary inflammation was to be feared, particularly pleurisy or pneumonia, and bleeding was the most likely means to prevent its developement. But all the cases were not so well marked. Sometimes a case of general plethora was masked under the deceptive appearance of weakness and prostration: the face was pale, pulse small, heat of skin moderate. Here every symptom tended to lead the practitioner astray. In such difficult cases, a careful examination of the heart and lungs often furnished valuable information. If the contractions were tumultuous, and the pulsation of the heart powerful, and if the respiratory murmur was feeble and oppressed, and without any other modification, M. Desormeaux had recourse to a tentative bleeding (*saignée explorative,*) and it was repeated, or not, according to the effect produced, and the appearance of the blood. General bleeding was sometimes found useful in that short period of inflammatory action which usually precedes the developement of typhoid symptoms, although this variety of the inflammatory form of the disease, being less pure in its nature, and less decided in its progress, was more safely treated by local bleeding. The employment of general bleeding in these cases required great prudence. It could not be used beyond the first days, or often even after the first few hours; and, however intense the pain, it was inadmissible the moment typhoid symptoms appeared.

*Of local Bleeding.* The employment of local bleeding by means of leeches, may be considered, particularly in the disease of which we are treating, as a modern discovery; for, although it was not entirely neglected by the ancients, it is so differently employed in the present day, that it is in fact a new method of treatment. Of all the means employed in the puerperal diseases which we have witnessed, none was more frequently applicable, or followed by more favorable effects, than local bleeding by leeches. This practice was adopted in acute inflammation of the peritoneum and uterus, where there was strong general reaction; and M. Desormeaux in such cases often employed it after venesection. In the transitory and often ephemeral inflammations which usually precede the developement of typhoid symptoms, in which it was had recourse to from the very commencement, its employment was much more frequent than general bleeding. The leeches were always applied to the belly, and as many as forty or sixty, and they were often repeated on the same and following day, if the disease was not evidently checked. It very frequently happened that more than two hundred were applied to a patient in the space of thirty-six or forty-eight hours. When the leeches dropped off, the patient was put into an emollient hip-bath, and the belly was then covered with a large poultice, to keep up the bleeding. Each of these local bleedings produced as abundant a flow of blood as could have been drawn by venesection, but the subsequent effects were not at all similar. Venesection, by unloading the large vessels, usually produced a rapid amendment of the general inflammatory symptoms, without, however, always sensibly influencing the local malady. Bleeding by leeches, on the contrary, had a constant and well-marked influence upon the local pain, and the general effects were remote and secondary. The one produced rapid debility; the other was easily borne, even by the most feeble women. In some rare cases, where the powers of the constitution had been oppressed by the violence of the inflammation, local bleeding was followed by strong inflammatory reaction, which it was necessary to combat by venesection; but more commonly, after the application of leeches, there was a remarkable and immediate alleviation of pain, and a suspension of the disease, during which the pulse became soft, the heat of the skin and agitation diminished, perspiration broke out, and even the lochia reappeared. In most cases this calm was but momentary. The inflammation, being thus attacked only in its effects, was quickly reproduced, and it was necessary either to have recourse to general bleeding or to other means for the purpose of effectually conquering it. When effusion and suppuration were formed, and especially when any of those symptoms were present which indicated the absorption of pus, M. Desormeaux carefully abstained from the use of leeches, as he knew well from experience that no benefit could then be derived from such means, and that, on the contrary, it would probably be prejudicial. In this state he had recourse to other remedies, and particularly

*Mercurial Preparations.* Mercurial frictions upon the abdomen and internal part of the thighs, alternately, were usually preferred. Two or three ounces of strong mercurial ointment were rubbed in each day. Each friction, of two drachms, was regularly employed every one or two hours. The integuments covered with the ointment were cleansed every day with oil of sweet almonds, that their suppleness and permeability might remain. M. Desormeaux often combined the internal administration of calomel with the external use of mercury; but this mode of practice, which was well adapted to cases where there was constipation, or even moderate diarrhœa, was not applicable to the more numerous affections which were attended by abundant alvine evacuations. Eight or ten grains of calomel were generally given daily, combined with opium or extract of hyoscyamus, by which the digestive organs were rendered less susceptible to the action of the calomel, and at the same time the abdominal pains were relieved. This treatment was rarely practised from the commencement of the disease; but still our opinion of its propriety was much more guided by the character of the malady, than by the time which had elapsed since its invasion. As a general proposition, it may be said that, as soon as the primary symptoms of inflammation had given place to others indicative of effusion, suppuration, and especially of the absorption of pus, mercurial frictions were properly and usefully applied. This means was almost always preceded by general or local bleeding; but we must not fall into the error which some have committed, of supposing that the favorable result which followed the use of mercurials was attributable exclusively to the abstraction of blood, for the former were only employed because the latter had failed. The efficacy of the mercurial treatment appears then to be evident; and the more so as, when the disease had arrived at the point in which it was employed, death almost inevitably resulted under any other mode of treatment.

Much difference of opinion still obtains respecting this practice. Many physicians doubt its merits, and very wisely wait for additional facts before they either adopt or reject it. We offer to their notice, therefore, the results of M. Desormeaux's experience. But some practitioners are decidedly opposed to it, because it does not agree with their preconceived theoretical opinions. It is not our intention to discuss, *à priori*, a question which experience can alone determine; but still we may observe that such a mode of argument is unjustifiable. Theories must not be opposed to facts, of which they should be an exact and rigorous expression; and when every fact is not included in them, they must contain some inherent fault. But, besides this systematic obstinacy, we find a blind enthusiasm which adopts the treatment exclusively and rashly. The employment of mercurials is rejected by some as absurd and dangerous, while it is lauded as a specific by others, who appear to forget that their exaggerated eulogiums are calculated to injure the reputation of even our best remedies. The latter contend, and truly too, that the facts they adduce are incontrovertible; but from these insufficient data they hasten to draw *general* inferences, and hence their error. In their hands, we doubt not, mercurial frictions have frequently succeeded; but, if the circumstances under which they pursued the practice no longer exist, if the atmospheric constitution is changed or modified, if the malady which had presented itself to them in a sporadic form should become epidemic, the result will be very different. We have often found mercurial frictions successful, but they have more frequently failed: but we should not therefore be justified in abandoning a remedy which sometimes succeeds when all others fail, in cases where the death of the patient is almost inevitable.

To confirm our opinion of the efficacy of mercurial frictions, we shall detail a few cases which show the effects of this treatment, and, to a certain extent, its mode of action.

*Cases of Puerperal Fevers cured by Mercurial Frictions, followed by Salivation.*

Case I. St. Den . . ., æt. thirty, of a nervous and weak habit, admitted in May 1829, and was favorably delivered of her first child in July. The same night she had pains in the loins and hypogastrium. Next morning the pains had increased, and were attended with fever, headach, and some nausea. M. Desormeaux ordered fifty leeches to be applied: they drew blood freely, but produced little relief.

Third day, leeches were repeated.

Fourth day, amendment, and hopes of a speedy solution of the disease.

Fifth day, shiverings, and increased severity of all the symptoms. Forty leeches applied.

Sixth day: abdomen painful over its whole extent; face pale and anxious; limbs trembling; small and irregular pulse; involuntary discharge of brown fetid feces in large quantities. From this time bleeding was given up in this case, and mercurial frictions were substituted in the quantity of two drachms every two hours.

The seventh and eighth days, the frictions were continued, with no other appreciable effect than slight erysipelas; but the ninth, the diarrhœa ceased, and the pains were much diminished; countenance improved. Frictions discontinued.

Twelfth day: abundant salivation, which continued five or six days, and then yielded to astringent and opiate gargles, and oily laxatives.

Eighteenth day, the cure was complete, and the patient left the hospital.

Case II. Margaret B . . . ., æt. twenty-one, of good constitution, experienced, the second day after an easy labour, a prolonged shivering, which was quickly followed by violent pains in the hypogastrium and ardent fever. M. Desormeaux ordered fifty leeches to be applied immediately to the abdomen, and as many more in the evening. Symptoms unrelieved. The succeeding days their severity was increased, although fifty leeches had been twice more applied, and warm baths and calomel used.

Fifth day: several liquid stools, perspiration, and some amendment of the symptoms; but this improvement was temporary. In the night the woman was agitated and delirious. In the morning, the belly was very tender to the touch, and much swollen; face pale and sunken; skin hot and dry; respiration anxious, pulse rapid. Two ounces of mercurial ointment were now rubbed in every twenty-four hours: at first, without any very sensible benefit; but on the ninth day (the third of the mercurial treatment) the pains diminished, there was less depression, the pulse rose. The frictions were now discontinued.

The tenth day, the mouth was affected, and an abundant diarrhœa came on; a large quantity of puriform discharge flowed from the vagina. From this time the improvement was evident. The salivation increased much the following days, and was accompanied by a thick exudation on the tongue and internal membrane of the cheeks. Astringent gargles soon relieved these symptoms.

The patient quitted the hospital on the seventeenth day: she was pale, weak, and bloated, but out of danger. She had latterly felt a fixed pain in the muscles of the arm, and the formation of a deep-seated abscess was suspected.

There is a great analogy between these cases: intense inflammatory symptoms, rather local than general, were in each ineffectually opposed by vigorous bleeding. A calm occurred, it is true, but of that insidious nature which, as we have before remarked, often precedes suppuration. Very shortly new symptoms appeared, of a more formidable and very dangerous character. Mercurial frictions were only then employed, and we cannot reasonably refuse to them the merit of the cure. It is not to be doubted that the local abstractions of blood were of some service: if they did not entirely dispel the vascular congestion, it is at least probable that they diminished it, and thus paved the way for the favorable effects of the mercurial frictions. It is to be remarked that this means is not followed by the prompt and instant relief which is usually obtained from bleeding or leeches. Its beneficial effects were not manifested until after some days of painful uncertainty. The simultaneous occurrence of salivation, diarrhœa, and abundant flow of the lochia, must not pass unnoticed, and also the coincidence of these various circumstances with the cure of the disease. We shall subsequently, however, dwell more at length upon this important point.

### *Puerperal Fevers cured by Frictions, followed by Salivation and profuse Sweating.*

Case I. Dub . . ., ætat. twenty, nervous and irritable, had, on the third day after a favorable and first labour, all the symptoms of intense metro-peritonitis. During the first days of the attack, the following means were successively employed. Fifty leeches were twice applied to the hypogastrium; hip baths, emollient poultices, and other secondary remedies likely to fulfil the general indication. No benefit was derived. To the violent pains, vomiting and fever, which appeared at first and still continued, were added on the fourth day, copious diarrhœa, swelling of the abdomen, anxiety, great depression of the pulse, and a certain appearance of prostration, which appeared to indicate the presence of pus in the blood-vessels.

M. Desormeaux now substituted for the local bleedings, mercurial frictions, in the same quantities and manner as above mentioned. The third day of their employment, (seventh of the attack,) the abdominal pains diminished; the lochia reappeared, but were puriform and scanty; alvine evacuations less frequent. The frictions were now discontinued.

Eighth day: wandering pains in the limbs.

Ninth: profuse and almost constant sweating, accompanied by decided feelings of improvement and calm sleep.

Tenth: moderate salivation, which lasted four days; after which the patient left the hospital quite convalescent.

Case II. Henri . . ., æt. twenty-seven, of good constitution, was favorably delivered of her first child the 4th of November. The same day she had symptoms of metro-peritonitis, which increased to a great degree of violence on the following days. Ipecacuanha from the beginning; 210 leeches in four days; oily laxatives, hip baths, and emollient poultices, were the means used by M. Desormeaux, and with much advantage. But on the eighth day shiverings returned, and pain; the belly swelled, and fluid was evidently contained in its cavity; skin dry and hot, pulse small, frequent, and feeble.

Mercurial frictions were now begun; two ounces of ointment in eight applications each day. The second day of this treatment the patient was agitated and delirious. On the third, she was calm and sensible of improvement. An emulsion of castor oil was given to act upon the bowels, and the next day she had several liquid stools, and copious and long-continued sweating: sensibility of the abdomen was now much diminished, and on the fifth day entirely disappeared, at which time profuse sweating again occurred more abundantly than before, together with several free motions. The frictions were discontinued. On the sixth day the lochia reappeared, and the mouth was affected: salivation increased, and did not cease until the twelfth day, when the patient left the hospital cured.

Case III. *Profuse sweating; salivation very slight.* Brun . . ., æt. thirty, in good health, was attacked, the third day after her first labour, with metro-peritonitis, accompanied by violent inflammatory symptoms. The disease was opposed from its commencement by bleeding, and on the following days by two applications of fifty leeches each. On the sixth day the severity of the symptoms was diminished, but still there remained in the iliac fossa acute pain and deep-seated swelling, for which twenty leeches were applied, and some mercurial frictions, of a drachm each.

Tenth day: shivering.

Eleventh: severe pain and swelling over the whole of the belly; nausea, difficulty of breathing, and great prostration. M. Desormeaux now began the use of frictions in the quantity of two ounces in eight applications, and prescribed an oily mixture.

Twelfth: irregularity of pulse, and so much exhaustion that the patient with difficulty articulated a few words. Blisters were applied to the legs to produce general excitement. Thirteenth, she rallied a little.

Fourteenth: much better, and slept well during a part of the night.

Fifteenth: pains disappeared; a discharge of puriform lochia, and several stools procured by two ten-grain doses of calomel. The ointment was reduced to half an ounce, and the next day it was discontinued altogether.

Seventeenth: gums slightly, and but for a short time, affected. Profuse sweating occurred, and lasted for ten days, but still the patient gradually recovered her strength.

Thirtieth day, in the substance of the leg, a deep-seated swelling was detected. A collection of pus formed, and was soon discharged externally: the wound soon healed, and the patient left the hospital, in a very good condition, on the forty-first day.

In these cases, as in the two first, mercurial frictions were employed immediately, when the inflammatory symptoms gave place to those more serious signs indicative of the formation, and often of the absorption, of pus. From this period evacuation of blood must be useless, and even injurious: it would only diminish the remaining powers of the constitution, which are so much required to resist the injurious consequences of suppuration, or purulent absorption. The pus cannot be evacuated in these instances, as in external phlegmon; and its presence in the substance of various organs is incompatible with the due exercise of their functions. We have then to assist in its elimination by our therapeutic agents; and it appears that mercury is here a powerful auxiliary. At present, indeed, this opinion is hypothetical, but an attention to the various phenomena which follow the employment of mercurials confirms it to a certain extent. Authors, who have employed mercurial preparations in the treatment of puerperal fevers, have all observed that the appearance of salivation is almost always coincident with an amendment of the general symptoms; but they have passed over certain other, and not less remarkable facts, which were observed in the preceding cases. In most of these instances, salivation was accompanied by a discharge of the lochia, abundant alvine evacuations, or profuse and long-continued perspirations, followed by decided improvement. From these occurrences, the convalescence of the patient was so far from being procrastinated, that they appeared to tend towards the re-establishment of strength and health. Sometimes these various phenomena occurred simultaneously in the same individual; at others they happened separately, and in succession; and often they appeared reciprocally, to supply the place of each other. Thus, in the last case, almost immediately that the mouth became affected, profuse perspiration supplied the place of salivation; and in almost every instance where salivation did not occur, some other supplementary symptom was remarked. It is probable that these different organic actions are not merely destined for the evacuation of purulent secretions, but that they tend also towards the elimination of the mercurial particles, which have been shown by recent chemical analysis to circulate unchanged in the animal fluids, and to be deposed in the substance of different organs.* The art of the physician consists, therefore, not in opposing these various phenomena: by so doing, he would be contending with necessary symptoms. He must, on the contrary, assist and maintain them, and guide their progress. This appears to us to be the true system of physiological medicine, and it is that which M. Desormeaux pursued. To keep up perspiration by warm coverings and vapour baths, to provoke evacuations from the bowels by oily laxatives, to support the powers of the economy by light infusions of bark or calumbo, and never to oppose the above symptoms, except when they threaten to pass beyond a desirable extent, should be our constant endeavour.

(To be continued.)

* London Med. and Phys. Journal, September 1830, p. 202.

